# Turbid Peritoneal Fluid

**DOI:** 10.5811/westjem.2016.1.29444

**Published:** 2016-03-02

**Authors:** Samuel L. Burleson, Henry E. Wang

**Affiliations:** University of Alabama School of Medicine, Department of Emergency Medicine, Birmingham, Alabama

## CASE

A 58-year-old female with a past medical history of hepatitis C virus-induced cirrhosis presented to the emergency department with three days of increasing abdominal pain, chills, and nausea and vomiting. Abdominal physical examination revealed gross ascites with fluid wave. Diagnostic paracentesis resulted in the aspiration of approximately 60mL of white turbid peritoneal fluid (Figure).

## DISCUSSION

The differential diagnosis of turbid peritoneal fluid includes spontaneous bacterial peritonitis, chylous ascites, and pseudochylous ascites. Spontaneous bacterial peritonitis is suggested by a predominance of polymorphonuclear cells, a positive Gram stain or a positive culture.^1^ Chylous ascites refers to increased concentration of triglycerides (>200mg/dL)^2^ in the peritoneal fluid, typically the result of traumatic lymphatic obstruction, tumor, tuberculosis, filariasis, congenital abnormalities or nephrotic syndrome.^1^,^2^ Cirrhosis may cause up to 11% of atraumatic chylous ascites.^3^ Pseudochylous ascites results from degeneration of leukocytes or tumor cells without high levels of triglycerides or active infection.^4^ Chylous and pseudochylous ascites may be differentiated by triglyceride levels.^1^

Given the patient’s established history of chronic liver disease, the inpatient team focused on spontaneous bacterial peritonitis as the potential etiology for the turbid peritoneal fluid. The patient was treated empirically with antibiotics. Formal abdominal ultrasonography affirmed a cirrhotic liver with large volume ascites and no evidence of mass. Peritoneal fluid cultures were negative for bacterial growth, suggesting that the fluid represented sterile ascites. Triglyceride assays were not performed. Repeat paracentesis on hospital day 3 revealed straw-colored peritoneal fluid. The patient was discharged home on hospital day 4 after clinical improvement.

## Figures and Tables

**Figure:**
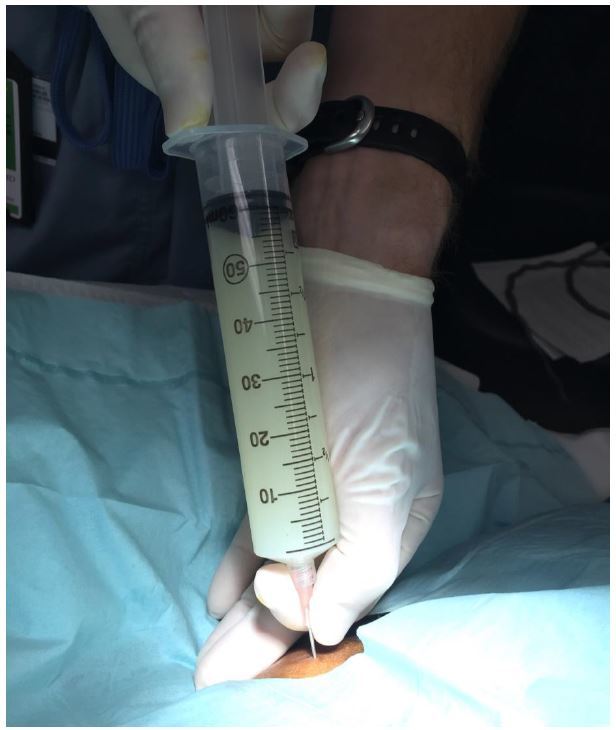
Peritoneal fluid aspirated from diagnostic paracentesis.
